# Preoperative information burden among parents of children with congenital heart disease: a qualitative study of information access, understanding, and decision-making

**DOI:** 10.3389/fpubh.2026.1965200

**Published:** 2026-10-12

**Authors:** Yuan Hu, Jing Tian, Mei Teng, Yuan Li, Hong Zheng

**Affiliations:** 1 Department of Pediatric Cardiology Nursing, West China Second University Hospital, Sichuan University, Chengdu, China; 2 West China School of Nursing, Sichuan University, Chengdu, China; 3 Key Laboratory of Birth Defects and Related Diseases of Women and Children, Sichuan University, Ministry of Education, Chengdu, China

**Keywords:** congenital heart disease, decision-making, family-centred care, health communication, health information seeking, healthcare access, parents, qualitative research

## Abstract

**Background:**

Parents of children with congenital heart disease must understand complex clinical information, navigate referral and practical barriers, and make treatment decisions on behalf of their child. Information burden may therefore reflect not only information quantity but also whether information is understandable, trustworthy, accessible, and actionable.

**Methods:**

A qualitative descriptive study was conducted at a tertiary paediatric cardiac centre in China. Twelve parents completed individual face-to-face semi-structured interviews. Purposive sampling sought variation in education, residence, and treatment pathway. Sample adequacy was guided by information power, and recruitment ceased when later interviews no longer altered the main analytic categories or theme boundaries. Data were analysed using the Framework Method, combining within-case timelines with cross-case comparison. Reporting followed the Consolidated Criteria for Reporting Qualitative Research (COREQ).

**Results:**

Four interacting themes were developed: threat before understanding; navigating fragmented information pathways; difficulty translating information into child-specific decisions; and information shaping family adaptation and treatment decisions. Parents described technical language, conflicting recommendations, changing findings, non-comparable digital stories, referral barriers, and cost uncertainty. They valued staged plain-language explanations, visual and written materials, explicit monitoring thresholds, a reliable route for clarification, verified digital information, and practical navigation.

**Conclusion:**

Preoperative information burden was driven less by information volume than by difficulty translating fragmented clinical, digital, and practical information into a trusted, child-specific course of action. Public health and paediatric services should treat communication, health-information access, and navigation as connected parts of a family-centred pathway rather than as isolated educational tasks.

## 1 Introduction

Congenital heart disease (CHD) affects approximately nine in every 1,000 live births and remains the most common congenital anomaly worldwide (1). Improved survival has created a growing population of children who require specialist assessment, surgery or transcatheter intervention, surveillance, and long-term partnership with families (2). Before intervention, parents must understand changing findings, consent to treatment, recognise deterioration, and coordinate travel, work, finances, and family responsibilities on behalf of a child who cannot assume those decisions.

Receiving a CHD diagnosis is often experienced as an immediate threat to the child’s life and future. Parents move rapidly from shock toward an urgent need for explanations that make the condition manageable (3). Distress is shaped by disease severity, uncertainty, and family resources (4), while caregivers approaching congenital heart surgery describe concerns about survival, intensive care, pain, recovery, and their own ability to protect the child (5). Across paediatric cardiac services, communication, involvement, and continuity are recurrent determinants of caregiver experience (6).

The information environment surrounding CHD extends far beyond a single consultation. Parents search, filter, remember, translate, and compare information; judge whether other families’ experiences are relevant; and relay an agreed account to relatives. Online searching and social media can fill gaps between clinical encounters but expose parents to uncertain source quality and highly salient stories from children with different anatomy and risk (7, 8). In China, parents of children with CHD move among hospital platforms, search engines, short-video applications, artificial-intelligence tools, and peer networks while navigating local and specialist care (9). Families outside specialist centres must also understand referral routes, travel, accommodation, and affordability, and these practical constraints can shape when and where care is sought (10, 11).

Health information overload is not simply a matter of volume: complexity, inconsistency, emotional arousal, and uncertainty about relevance can make even limited information difficult to use (12). Uncertainty management theory further suggests that people may seek more information, preserve a reassuring interpretation, or avoid information that feels unmanageable (13). From a public health perspective, health literacy concerns people’s capacity to access, understand, appraise, and use information in ways that support health decisions (14). For parents of children with complex health needs, these demands are intensified because caregivers must act as proxy decision-makers and navigate multiple services (15). CHD-specific commentary has similarly argued that health literacy is shaped by stress, complexity, access, and the organisation of communication rather than by reading ability alone (16).

Existing interventions show that structured education, responsive support, and digital tools can improve knowledge and engagement in congenital heart care (17–19), and digital interventions have broader potential to improve parental health knowledge and health-seeking behaviour (20). However, less is known about how parents themselves experience the preoperative information pathway, why information becomes difficult to translate into a child-specific decision, and how communication and service access interact to create or reduce burden. Addressing this gap is relevant to health communication, family health literacy, equitable access to specialist paediatric care, and the design of family-centred public health services. This study therefore explored parents’ preoperative information work, how information became burdensome, and which forms of communication and navigation support they considered useful before cardiac surgery or transcatheter intervention.

## 2 Materials and methods

### 2.1 Design and reporting

A qualitative descriptive design was used to produce a clinically grounded account in language close to participants’ descriptions while allowing comparison across the preoperative pathway (21). Reporting followed the Consolidated Criteria for Reporting Qualitative Research (COREQ) (22). The completed COREQ checklist is provided as supplementary material.

The study was situated within a pragmatic qualitative-descriptive orientation. Parents’ accounts were treated as contextually situated descriptions of information and decision-making, and the analysis aimed to produce a clinically useful account close to participants’ language rather than to generate formal theory or infer causal relationships.

### 2.2 Setting, participants, and sample adequacy

The study was conducted from 1 March to 30 June 2026 at a tertiary paediatric cardiac centre in Chengdu, China. Purposive sampling sought variation in parental education, residence, disease complexity, and planned treatment pathway. Eligible participants were adults aged 18 years or older who were a parent or primary caregiver of a child with confirmed structural CHD and had sought or received information before cardiac surgery or transcatheter intervention. Parents were excluded when acute distress or communication difficulty prevented meaningful participation. Cardiomyopathy and isolated tachyarrhythmia were outside the study scope.

No parent was excluded at eligibility screening because of acute distress or communication difficulties (0 in each category). Consequently, no support or referral was required as a result of exclusion on either of these grounds. The criterion concerning distress applied to eligibility for starting an interview; a separate participant later chose to discontinue an interview because some questions were emotionally painful, as reported in Section 3.1.

Sample adequacy was guided by information power rather than a predetermined numerical target (23, 24). The research question was focused, the participant group was specific, interviews addressed a common preoperative pathway, and data collection and analysis proceeded iteratively. Recruitment ceased when later interviews no longer altered the main analytic categories, theme boundaries, or explanatory relationships in the developing framework. This was treated as a transparent judgement of adequacy for the focused question rather than as a claim that every possible parental experience had been exhausted.

### 2.3 Data collection

Potential participants were identified and contacted by Yuan Hu through the inpatient ward. Before consent, participants were informed that the interviewers were professional nurses and members of the research team and were told the study purpose, voluntary nature of participation, confidentiality arrangements, and intended use of anonymised data. Interviews were conducted by Hong Zheng or Yuan Li, both female registered nurses and senior clinical nurses with formal qualitative-interview training and previous interviewing experience. Hong Zheng held a master’s degree and Yuan Li held a bachelor’s degree. Neither interviewer had previously participated in the clinical care of the children or families interviewed.

Each participant completed one individual face-to-face semi-structured interview in Chinese in a private, quiet room within the department. No non-participants were present. Interviews lasted approximately 30 min and were audio-recorded with permission. No formal field notes were taken. Within 24 h, Mei Teng and Jing Tian transcribed each recording verbatim, checked the transcript against the audio, and removed identifying information. The guide began with the child’s diagnostic and treatment history and then explored information needs and sources, conflicting or difficult-to-understand information, emotional and decisional effects, coping responses, and preferred support. Probes elicited concrete events rather than general opinions. The complete interview guide is provided in Supplementary file S1.

### 2.4 Analytic scope and data analysis

The analytic dataset covered diagnosis, observation, repeat assessment, referral, hospital and specialist selection, treatment decision-making, admission, and waiting for cardiac surgery or transcatheter intervention. Ten parents were interviewed before treatment. Two interviewed after treatment contributed detailed retrospective accounts of the preoperative period; postoperative material was used only when it clarified how they evaluated information received before treatment. Because only two interviews were conducted after treatment, interview timing was not used to define analytic subgroups and no formal comparison by interview timing was undertaken. One completed face-to-face interview was excluded before formal coding because the parent’s answers were predominantly outside the study’s information-focused research questions and the transcript contained insufficient material on preoperative information seeking, understanding, appraisal, or decision-making to support the focused analysis. Completion of a face-to-face interview did not, in itself, establish analytic relevance; this decision concerned the content of the completed transcript rather than the participant’s willingness to take part.

The Framework Method was selected because interviews addressed comparable domains while retaining the clinical and temporal sequence within each family (25). A case summary reconstructed each child’s diagnostic history and the parent’s information timeline. Jing Tian and Mei Teng, both female registered nurses with master’s degrees, coded the Chinese transcripts and developed preliminary categories, subthemes, and themes. Codes captured information sought, sources, credibility and relevance judgements, barriers to understanding or use, emotional and decisional consequences, coping responses, and requested support. Unresolved coding or thematic issues were discussed with Yuan Hu, a male registered nurse with a master’s degree, who made the final analytic decision.

The developing framework was applied across the dataset and charted manually in a participant-by-domain matrix; no qualitative data-analysis software was used. Within-case and cross-case comparisons examined recurring patterns, contextual differences, and divergent accounts. Theme boundaries were checked against complete transcripts, and code frequency was not treated as evidence of importance. Selected quotations were translated into English by Yuan Hu and Yuan Li after theme development and checked against the Chinese text; uncertainties were discussed with Hong Zheng.

### 2.5 Rigour and reflexivity

Trustworthiness was supported through coding by two researchers, repeated reference to complete Chinese transcripts, a decision log documenting changes to the analytic framework, and retention of divergent accounts, including limited searching and information avoidance. Reflexive notes considered how the interviewers’ clinical roles and prompts concerning information overload could influence responses and interpretation. Although neither interviewer had provided direct clinical care to the participating children or families, both were nurses within the same institution, and the research team recognised that Hong Zheng’s senior nursing role could create perceived authority and influence how freely parents expressed criticism of communication or care. This possibility was explicitly considered during reflexive discussions and interpretation of the data. Hong Zheng, a chief nurse and paediatric cardiology head nurse with more than 30 years of paediatric nursing experience, reviewed the framework for clinical coherence. Her dual role as interviewer and clinical reviewer was considered during team reflexive discussions. Participants could not be recontacted for transcript return or member checking. In the absence of member checking, interpretive credibility was supported through dual coding, repeated checking against complete transcripts, a documented analytic decision trail, examination of divergent accounts, and reflexive team discussion.

### 2.6 Ethics statement

The Medical Ethics Committee of West China Second University Hospital, Sichuan University approved the study (Approval No. 2025453). All participants provided written informed consent before data collection, including permission for audio-recording and publication of anonymised quotations. Participation was voluntary, withdrawal did not affect the child’s care, and participants were assigned codes P1–P12.

## 3 Results

### 3.1 Participant characteristics

Sixteen parents were recruited. One parent withdrew during the interview, explaining that some questions brought back painful experiences and that they did not wish to continue; the request to stop was respected. Two were excluded because their children had cardiomyopathy or isolated tachyarrhythmia rather than structural CHD, and one completed interview was excluded before coding because it provided insufficient relevant content. The final sample comprised 12 parents: nine mothers and three fathers. Mean age was 32.4 years (SD 6.9; range 21–40). Five had college or university education and five had junior high school education or below. Families lived in rural areas, county towns, and cities. Ten interviews occurred before treatment and two afterwards. Participant characteristics are presented in Table 1. Four themes and 15 subthemes were developed (Table 2). Figure 1 summarises their relationships without implying a causal or uniform temporal sequence.

**Table 1:** Participant characteristics (*N* = 12).

Characteristic	Category	*n* (%) or mean ± SD
Parent age, years	-	32.4 ± 6.9
Relationship to child	Mother	9 (75.0)
	Father	3 (25.0)
Education	Primary school or below	1 (8.3)
	Junior high school	4 (33.3)
	High school/technical secondary school	2 (16.7)
	College/university	5 (41.7)
Residence	Rural area	5 (41.7)
	County town	5 (41.7)
	Urban area	2 (16.7)
Child sex	Male	9 (75.0)
	Female	3 (25.0)
CHD complexity	Simple	5 (41.7)
	Complex	5 (41.7)
	Not classified in available data	2 (16.7)
Interview timing	Before surgery/intervention	10 (83.3)
	After surgery/intervention	2 (16.7)
Treatment pathway	Cardiac surgery	7 (58.3)
	Transcatheter intervention	4 (33.3)
	Undetermined at interview	1 (8.3)

Note. Interview timing refers to data collection relative to the relevant intervention. All participants contributed preoperative material. CHD, congenital heart disease; SD, standard deviation.

**Table 2:** Themes, subthemes, and analytic meaning.

Theme	Subtheme	Analytic meaning	Illustrative participants
Threat before understanding	Disease label before usable meaning	Clinical terminology conveyed danger without anatomy, prognosis, or next action.	P1, P11
	Spontaneous-closure message displaced monitoring logic	Hope was retained more readily than warning signs, review thresholds, or consequences of persistence.	P1, P2
	Communication framed urgency and possibility	The same diagnosis became catastrophic or manageable depending on the plan attached to it.	P5, P7
Navigating fragmented information pathways	Digital searching filled access gaps	Parents used search engines, social media, online consultations, and AI tools between encounters.	P3, P4
	Peer stories as experiential evidence	Other families supplied practical detail but were difficult to compare with the child.	P9, P12
	Service entry and cost as information work	Campus, appointment, referral, travel, and financial information shaped whether care was reachable.	P1, P7, P8
	Trust narrowed to a specialist team	A responsive specialist team stabilized interpretation and reduced further comparison.	P3, P7
Difficulty translating information into child-specific decisions	Technical density and poor retention	Parents could not reliably retain or relay dense explanations and consent information.	P9, P11
	Conflicting recommendations without conditions	Families saw incompatible conclusions but not the clinical contingencies behind them.	P2, P10
	Changing findings reset understanding	New abnormalities invalidated earlier assumptions and restarted searching.	P3, P6
	Avoidance and constrained access	Low information volume could reflect limited literacy, fear, or lack of access rather than low burden.	P6, P8
Information shaping family adaptation and treatment decisions	Sleep and emotional consequences	Threat monitoring and repeated checking entered everyday caregiving.	P1, P4, P9
	Redistribution of family information work	Searching, clinician communication, and emotional protection were divided between parents.	P1, P10
	Treatment timing and decision closure	Information contributed to delay, acceleration, or narrowing around one trusted team.	P1, P3, P10
	Staged and reachable support	Parents wanted information matched to the next decision and a legitimate route for questions.	P7, P11

Participant codes are illustrative and are not presented as frequency counts.

**Figure 1:**
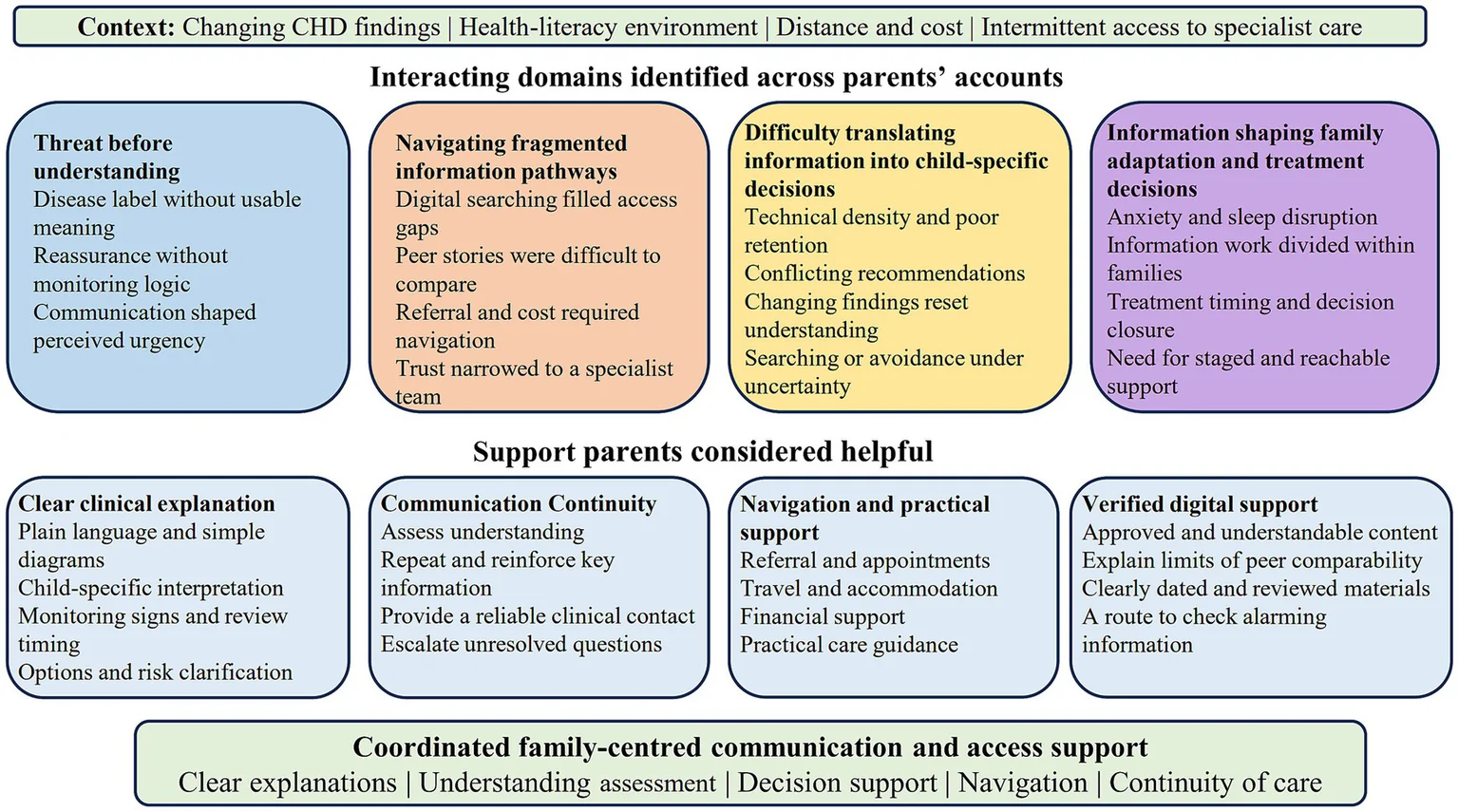
Preoperative information burden and family-centred communication and access support identified from parents’ accounts. The upper section presents four interacting domains developed from the interviews. The lower section summarises support parents considered helpful, including clear clinical explanation, communication continuity, navigation and practical support, and verified digital support. The figure does not represent a causal pathway or a uniform temporal sequence.

### 3.2 Threat before understanding

Parents often understood the diagnosis first as danger rather than as an anatomical or clinical problem they could explain. Technical labels conveyed seriousness but did not necessarily provide usable meaning.

“When they said there was a defect in the heart, we did not even know what congenital heart disease meant. I was frightened, but I still did not know what the disease actually was.” (P1)

Reassurance that a defect might close spontaneously offered hope and was retained more readily than warning signs, review thresholds, or the consequences of persistent shunting. Observation could therefore be interpreted as permission to wait rather than an active surveillance plan.

“Some children could grow out of it, so I thought it was better to wait until he was older. When the left side of the heart enlarged, I realised we could not keep waiting.” (P2)

The wording and practical direction attached to clinical communication shaped perceived urgency. Statements that a local hospital could not treat the child were heard as evidence of catastrophe when no onward plan followed, whereas specialist explanations that linked findings to a treatment plan made the diagnosis more manageable.

“The first doctor said, ‘Our hospital cannot treat this,’ and made it sound very serious. We were frightened. After the specialist explained what could be done, we gradually became calmer.” (P7)

### 3.3 Navigating fragmented information pathways

Parents used search engines, short-video applications, online consultations, hospital accounts, artificial-intelligence tools, and peer networks when specialist access was limited or questions remained after consultations. Searching was purposeful: parents were trying to create a workable explanation, estimate risk, and identify where the child should be treated.

“I tried every browser I could—Baidu, QQ and Doubao. I compared the information, although I did not know whether the data were accurate.” (P3)

Other families’ experiences supplied practical detail about intensive care, recovery, and repeat procedures, but parents lacked a reliable basis for deciding whether those stories were comparable with their own child.

“Some parents said the operation went very well. Others said the child stayed in ICU for a long time or needed another operation. The more I read, the more frightened I became.” (P9)

Entering specialist care was itself an information task. Families travelling to Chengdu had to identify the correct campus, department, specialist, appointment route, referral sequence, reimbursement pathway, and accommodation while fearing deterioration.

“There are several hospitals called West China and more than one campus. At first, even knowing what to type into the map was difficult.” (P1)

Broad searching often narrowed once a specialist team linked findings to action and remained reachable. Some parents deliberately stopped further comparison because every new opinion risked reopening a difficult decision.

“Once we had chosen this team, I felt we should stop changing our minds. Otherwise every new opinion would make us start again.” (P3)

### 3.4 Difficulty translating information into child-specific decisions

Information became burdensome when parents could hear or collect information but could not connect it to what should happen for their child. Investigations and consent discussions introduced multiple unfamiliar concepts in a short period; apparent agreement in the consultation sometimes masked poor retention.

“The doctor said many things and I nodded. When I went back and my family asked me, I could not say clearly what any of it meant.” (P11)

Conflicting recommendations were especially difficult when the clinical conditions underlying them were not visible to families. Parents heard advice to wait or intervene, operate early or allow the child to grow, and choose surgery or transcatheter treatment, but were left comparing conclusions rather than reasoning.

“Some doctors said it was fine to do the operation when the child was older; others said the earlier the better. When one doctor said this and another said that, I became confused.” (P2)

New findings could invalidate an explanation that had only recently become stable. Airway, valve, or pulmonary-pressure abnormalities were experienced not as additional facts but as reasons to rebuild understanding and restart searching.

“Before the CT, all our attention was on the pulmonary artery sling. When the airway narrowing appeared, I started searching again because the operation was no longer as simple as we had thought.” (P3)

High information volume was not necessary for burden. Limited digital confidence, lack of usable search terms, or fear also produced avoidance and dependence on a single authority.

“I only used Baidu, but I did not find anything I could understand. Sometimes I just sat at home and cried.” (P8)

### 3.5 Information shaping family adaptation and treatment decisions

Repeated checking entered everyday caregiving through sleeplessness, repeated viewing of procedural videos, and heightened attention to symptoms mentioned online.

“He told me it was a simple operation, but he searched for information all night. He was still searching the day before surgery.” (P4)

Information work was also divided within families. One parent often searched and communicated with clinicians while the other focused on feeding, childcare, or emotional containment. This could protect a more anxious parent but also create tension over whether further searching was helpful.

“We argued several times because we were both frightened. It was not that we wanted different things; we were both trying to do what was best for the child.” (P1)

Information influenced treatment timing and decision closure. Messages about spontaneous closure contributed to delayed reassessment, whereas stories of infection or deterioration accelerated referral. A child-specific explanation from a trusted specialist often ended prolonged comparison.

“When the doctor explained why this was the right time and what the risks meant for our child, we decided to proceed.” (P10)

Parents did not ask for all available information at once. They wanted plain-language explanations and a simple diagram at diagnosis, explicit monitoring thresholds during observation, child-specific reasoning about options during decision-making, and practical preparation after admission. They also valued written prompts, repeated explanation, a legitimate contact who could verify alarming information, and help reaching the correct service (Table 3).

**Table 3:** Stage-specific information needs and parent-preferred support.

Preoperative phase	Decision facing the parent	Information failure described	Parent-preferred support
Diagnosis and observation	What is the defect? How serious is it? Can it close? What change should prompt review?	Threatening terminology, vague reassurance, and no explicit monitoring thresholds.	Plain-language explanation, a simple diagram, warning signs, review timing, and a written next step.
Treatment decision and referral	Does the child need treatment now? Which procedure and centre are appropriate? Can the family afford and reach care?	Conflicting advice, non-comparable peer stories, unclear referral routes, and financial uncertainty.	Child-specific reasoning for timing and options, verified referral guidance, and financial-resource information.
Admission and preparation	What do the tests mean? What are the anaesthetic, surgical, and intensive-care risks? What must the family prepare?	Dense information, rapid consent discussions, late new findings, and repeated searching while waiting.	Sequenced explanation, teach-back, a one-page checklist, verified digital material, and a route for unresolved questions.

“Tell us what is most important now, what we should do next and when we need to come back.” (P11)

## 4 Discussion

### 4.1 Information burden as a problem of actionability and the health-literacy environment

The central finding was that preoperative information burden reflected difficulty translating fragmented information into a child-specific course of action more than information volume alone. Parents integrated diagnostic labels, serial findings, professional recommendations, peer stories, referral instructions, and financial constraints while carrying responsibility for the decision. This extends work showing that parental understanding develops by connecting professional explanation with observations of the child (26). In a public health framing, the issue is not simply whether parents possess sufficient individual knowledge, but whether the surrounding health-literacy environment makes information and services findable, understandable, appraisable, and usable (14–16).

This framing places information burden close to decisional conflict rather than simple information excess. In parents of children with congenital heart defects, lower decisional conflict has been associated with greater nurse support and clearer physician risk communication (27). A qualitative study of surgical decision-making in Taiwan likewise described rapid accumulation of knowledge, pervasive uncertainty, and trust in clinicians alongside a need for clearer communication (28). The practical implication is that information becomes useful when it reduces uncertainty about the next action, not merely when more facts are supplied.

The coexistence of repeated searching and information avoidance is also important. Searching could represent attempts to find a stable explanation, whereas avoidance could protect parents from material they could not interpret or act on. CHD coping research shows that parents move between problem-focused and emotion-focused strategies while managing stress (29). Neither response should automatically be labelled non-adherence. A more informative assessment is whether the family can explain the current plan, its rationale, the signs that would require reassessment, and where to obtain reliable clarification.

### 4.2 Communication and proxy decision-making

Reassurance about spontaneous closure was remembered more readily than the conditions attached to observation. Observation should therefore be communicated as an active contingency plan with review timing, warning signs, and thresholds for changing course. When genuine treatment options exist, decision aids can improve knowledge and risk perception (30), but their usefulness in CHD depends on connecting general evidence to anatomy, haemodynamics, age, symptoms, and local expertise. Teach-back is relevant because agreement during consultation does not establish that a parent can later explain the rationale and next step (31).

Parents also often decide as a family unit. In paediatric life-threatening conditions, parents describe differences in roles, information access, emotional responses, and opportunities to discuss choices together (32). Our finding that information work was divided within families suggests that communication should be designed for the family system rather than assuming that one brief encounter with one parent is sufficient. Where possible, both caregivers should have access to a shared explanation, written next steps, and a legitimate route for questions.

This has implications beyond the consultation room. Specialists remain responsible for diagnosis, indication, procedural alternatives, and child-specific risk; nurses can assess understanding and reinforce the plan; social and administrative services can address referral and financial barriers; and digital communication teams can maintain trusted resources. The public health contribution lies in treating these activities as a connected communication system that supports informed proxy decision-making across settings.

### 4.3 Navigation, continuity, and equity across the care pathway

Trust narrowed when a specialist team connected findings to action and remained reachable. Families travelling from rural or county areas also had to solve appointments, referral, transport, accommodation, reimbursement, and charitable-support questions. Evidence for paediatric navigation programmes remains limited, supporting explicit role definition and evaluation rather than assuming that a navigator alone will resolve access problems (33). Journey-mapping research shows that needs and service failures change across time and touchpoints (34), including across cardiovascular rehabilitation pathways (35).

Access and continuity therefore have an equity dimension. A qualitative study of critical CHD care in New Zealand showed that communication, access, cultural context, and coordination shaped families’ experiences and opportunities for equitable care (36). People living with CHD more broadly identify relationships and communication, access and coordination, discrete episodes of care, and psychological support as interdependent features of good care (37). In the present study, practical information about referrals, travel, reimbursement, and accommodation was part of the health-information burden because it determined whether specialist recommendations were realistically actionable.

For public health services, this suggests that information quality cannot be evaluated only by the accuracy of educational content. A technically accurate explanation may still fail if families cannot identify the correct service, return at the recommended time, afford travel, or access clarification when findings change. Communication quality, navigability, and service accessibility should therefore be considered together when designing support for families of children with complex chronic conditions.

### 4.4 Digital information as both access support and a source of uncertainty

Digital searching filled periods when clinical access was intermittent. Advising parents simply to avoid social media or artificial-intelligence tools would leave the access gap unchanged. Digital interventions can improve parental health knowledge and health-seeking behaviour, although evidence remains heterogeneous and relatively limited (20). A safer service response is to provide verified starting points, explain why peer experiences may not be comparable, identify review dates, and offer a route for checking alarming content. This requires organisational and informatics capability rather than relying on individual clinicians to correct misinformation after it has already influenced a family (38).

This approach is consistent with family-centred developmental care, in which parents value continuing involvement and relational support (39), and with evidence that CHD families experience clinical, caregiving, financial, and interpersonal pressures as overlapping burdens (40). Digital information should therefore be integrated with, rather than separated from, clinical communication and practical navigation. The goal is not to control what parents search for but to make reliable information easier to find, easier to interpret, and easier to connect to a child-specific plan.

### 4.5 Implications for family-centred public health and paediatric services

Recent cardiovascular literature supports combining individualised information with relational follow-up. Women with CHD reported greater safety when they were regularly followed, informed, involved, and supported by a multidisciplinary team (41). Person-centred cardiovascular care likewise requires personalised recommendations to remain aligned with individual needs, participation, psychosocial context, and equity (42). Although these studies concern different CHD populations, the principle is relevant to parents acting as proxy decision-makers: precision is only useful when families can understand, question, access, and act on it.

One potential extension of these findings is the development of clinician-supervised, human-centred Decision Support Systems (DSS) that target the actionability gap rather than simply increase information volume. Such systems could organise evidence-based information around the child’s diagnosis, current clinical findings, treatment stage, and family priorities; distinguish general education from child-specific recommendations; and present next-step options in a structured way. The NOVA-L framework, proposed for parental decision-making after life-limiting fetal diagnoses, illustrates how a digital DSS might combine evidence, care options, and family values within a professionally supervised interface; however, it was developed in a different clinical context and remains a conceptual framework rather than a validated CHD intervention (43). More directly relevant to paediatric cardiac care, the Heart OBServation app was co-developed with parents and clinicians to provide personalised information and guide parents in recognising deterioration and deciding when and whom to contact (44). In the preoperative CHD pathway, a comparable system could also integrate referral, appointment, financial, and travel navigation. Such tools should complement rather than replace clinician-parent communication, and future CHD-specific systems should be co-designed with families and evaluated for comprehension, decisional conflict, usability, equity of access, and the possibility of generating new forms of information burden.

For paediatric services, four priorities emerge. First, stage information around the next decision rather than deliver a comprehensive package at one encounter. Second, assess what parents can explain and apply, not simply what information has been delivered. Third, connect communication with referral, appointment, financial, and travel navigation so that the care plan is practically reachable. Fourth, integrate digital resources with a clear clinical verification route. These priorities are consistent with existing evidence that structured education and responsive digital support can improve knowledge and engagement in CHD care (17–19).

These findings should not be interpreted as evidence that every parent needs the same amount or type of information. Rather, services can reduce avoidable burden by making the pathway more health-literate: offering plain-language and visual explanations, making monitoring thresholds explicit, documenting a shared next step, providing reliable contact routes, and reducing navigation demands that fall disproportionately on families with fewer resources. Future intervention research should co-design these components with families and evaluate comprehension, decisional conflict, continuity, access, navigation delay, and unintended workload.

### 4.6 Strengths and limitations

The study captured first-hand accounts across variation in education, residence, disease complexity, and treatment pathway. Within-case timelines preserved links between information, appraisal, and decisions, while divergent cases prevented information burden from being equated with extensive searching. The analysis also connected clinical communication with digital and practical information work, which is important for understanding how service design shapes families’ ability to act on health information.

Several limitations require caution. The sample was small and drawn from a single tertiary specialist centre. Recruitment in this setting may have preferentially included families who had already succeeded in reaching specialist cardiac care and may therefore underrepresent families who remained within local services, encountered greater referral barriers, or were unable to access tertiary care. Although purposive sampling sought variation in education, residence, disease complexity, and treatment pathway, formal health-literacy and socioeconomic measures were not collected. The findings should therefore not be assumed to represent families with different levels of health literacy, digital access, financial resources, or familiarity with the healthcare system, and transferability to other regions, service configurations, and sociodemographic contexts should be considered cautiously. Two parents were interviewed after treatment and reconstructed their preoperative experiences retrospectively. Knowledge of the treatment process and outcome may have reframed how earlier uncertainty, communication, or decision-making was remembered. Because only two interviews were retrospective, the study was not designed to support a formal comparison by interview timing, and it cannot determine whether interview timing systematically influenced theme development. The interview guide named examples of information overload and may have increased their salience. No formal field notes were collected. Social desirability may also have influenced participants’ accounts because interviews were conducted by nurses from the same institution; despite the absence of a prior direct-care relationship, Hong Zheng’s senior nursing role may have discouraged some parents from expressing criticism of staff communication or care. Participants could not be recontacted for member checking, so interpretations were not returned to them for confirmation; credibility was instead supported through dual coding, repeated checking against complete transcripts, a documented analytic decision trail, examination of divergent accounts, and reflexive team discussion. The proposed service and digital decision-support implications should therefore be treated as hypotheses for family co-design and prospective evaluation rather than as tested interventions.

Although no parent was excluded at screening for acute distress or communication difficulties, these eligibility criteria could, in other settings, limit the inclusion of caregivers facing substantial emotional or verbal-communication barriers. In addition, one participant stopped an interview because some questions brought back painful experiences; their unfinished account was not included in the analytic dataset. The analytic sample may therefore provide less insight into experiences of parents for whom discussing the preoperative period is particularly distressing. The study cannot characterise the reasons for communication difficulties among excluded parents because there were no exclusions on that basis.

## 5 Conclusion

Preoperative information burden among parents of children with CHD arose when clinical, digital, and practical information could not be translated into a trusted, child-specific plan. The findings suggest that health communication, health-information access, and practical navigation are interdependent parts of family-centred care. Services should stage information around the next decision, assess understanding, make monitoring thresholds explicit, provide reliable routes for clarification, and help families navigate referrals and digital information. These strategies may reduce avoidable information burden and support more equitable proxy decision-making across complex paediatric care pathways.

## Data Availability

The raw data supporting the conclusions of this article will be made available by the authors, without undue reservation.
