# Supplementary material for: Preoperative information burden among parents of children with congenital heart disease: a qualitative study of information access, understanding, and decision-making
Source: Front Public Health. 2026 Oct 12;14:1965200. doi: 10.3389/fpubh.2026.1965200 (PMC13696496; doi:10.3389/fpubh.2026.1965200)
Supplement: Supplementary file 1 [file Data_Sheet_1.PDF]

Supplementary File S1. Semi-structured interview guide

Use of the guide

The guide was used flexibly. The sequence of questions was adjusted to the flow of each interview, and follow-up prompts were used when clarification or a concrete example was required. Participants were encouraged to describe their own experiences; there were no right or wrong answers.

Introductory statement

Thank you for agreeing to take part in this interview. This study aims to understand parents' experiences of obtaining, understanding, evaluating, and using information related to their child's congenital heart disease, particularly before surgery or transcatheter intervention. We are interested in situations in which information may have been excessive, complex, difficult to understand, inconsistent, or hard to judge. We would also like to learn what forms of professional and service support parents consider helpful. Please describe your own experiences as openly as possible. There are no right or wrong answers. With your permission, the interview will be audio-recorded. All information will be kept confidential and reported anonymously.

Core interview questions

1. Could you describe your child's experience from the first recognition or diagnosis of congenital heart disease to the present?

- When and how was the condition first identified?
- What important events occurred before the current admission or planned treatment?

2. During this period, what information did you most want to know?

- Why was this information important to you?
- Did your priority questions change as your child's condition or treatment plan changed?

3. Where did you usually obtain information about the condition, treatment, and care of your child?

- For example, healthcare professionals, hospital platforms, internet searches, social media, artificial intelligence tools, relatives, or other parents.

- Which sources did you use most often, and which did you trust most? Why?

4. Did you ever feel that there was too much information, that the information was too complex or difficult to understand, or that you did not know which source to trust?

- Could you describe a specific experience that you remember clearly?
- What made the information difficult to use?

5. Did you encounter differences or contradictions between information provided by doctors, nurses, online sources, other parents, relatives, or friends?

- How did you judge which information was applicable to your child?
- What did you do when different sources gave different advice?

6. At which stage did you experience the greatest information-related pressure?

- For example, at diagnosis, during observation and follow-up, when deciding whether or when to treat, when selecting a hospital or clinician, or while waiting for surgery or transcatheter intervention.
- What made that stage particularly difficult?

7. When information was extensive, confusing, or inconsistent, how did it affect you and your family?

- Did it affect your emotions, sleep, family relationships, or confidence in caring for your child?
- Did different family members respond to the information in different ways?

8. Did the information you received influence treatment or caregiving decisions?

- For example, whether or when to seek treatment, which hospital or clinician to choose, whether to continue observation, or how to care for your child.
- Could you describe how the information affected a particular decision?

9. How did you usually respond when information-related pressure became difficult to manage?

- Did you seek further information, repeatedly consult healthcare professionals, ask other parents, discuss the situation with family members, or temporarily avoid information?
- Which approaches helped, and which made the pressure worse?

10. Of the information provided by healthcare professionals, what was most helpful?

- What information was insufficient, unclear, or provided too late?
- Were there questions that you wanted to ask but did not know how, or did not have an opportunity, to ask?

11. If the hospital were to provide additional information support for parents, when and how should it be provided?

- At which stages would support be most useful?
- Would you prefer face-to-face explanation, written materials, diagrams, short videos, telephone, or online contact?
- What information should be included?

12. Is there anything important about obtaining, understanding, or managing information related to your child's condition that I have not asked about?

- Is there any experience or suggestion that you would like healthcare professionals or health services to understand?

Closing statement

Thank you for sharing your experiences. Before ending the interview, the interviewer briefly summarised the main points and invited the participant to correct, clarify, or add any information.

Supplementary Table S1. Proposed family-centred health communication and access-support framework derived from the findings

Domain	Proposed service response	Potential evaluation indicators
Health communication and understanding	Provide staged plain-language explanations and simple diagrams; document the child-specific rationale, monitoring thresholds, and next step; use teach-back or equivalent understanding checks.	Parent comprehension; unresolved questions; decisional conflict; repeat clarification needs.
Continuity and clarification	Provide a legitimate contact route for unresolved or conflicting questions, with clear escalation to the appropriate specialist.	Response time; continuity; specialist escalation; duplicate enquiries; after-hours workload.
Navigation and equitable access	Screen for referral, appointment, travel, accommodation, insurance/reimbursement, and charitable-support needs before they delay care.	Navigation delays; missed appointments; time to resolution; access barriers by residence or resource level.
Digital information environment	Provide verified, dated, understandable digital resources; explain limits of peer comparability; provide a route for checking alarming online content.	Use and comprehension of verified resources; misinformation-related enquiries; content review compliance.

Family-centred evaluation	Co-design communication pathways with parents and evaluate both family and service outcomes.	Confidence, comprehension, continuity, complaints, workload, and acceptability.
