# Supplementary material for: Preoperative information burden among parents of children with congenital heart disease: a qualitative study of information access, understanding, and decision-making
Source: Front Public Health. 2026 Oct 12;14:1965200. doi: 10.3389/fpubh.2026.1965200 (PMC13696496; doi:10.3389/fpubh.2026.1965200)
Supplement: Supplementary file 2 [file Data_Sheet_2.PDF]

COREQ (Consolidated Criteria for Reporting Qualitative Research) Checklist

Manuscript: Preoperative information burden among parents of children with congenital heart disease: a qualitative study of information access, understanding, and decision-making

Reference: Tong A, Sainsbury P, Craig J. Consolidated criteria for reporting qualitative research (COREQ): a 32-item checklist for interviews and focus groups. Int J Qual Health Care. 2007;19:349-357. doi:10.1093/intqhc/mzm042.

No.	COREQ item	Guide question	Reported in manuscript	Details
1	Interviewer/facilitator	Which author/s conducted the interview or focus group?	Materials and methods: Data collection	Hong Zheng or Yuan Li conducted each interview.
2	Credentials	What were the researcher's credentials?	Materials and methods: Data collection / Data analysis	Hong Zheng held a master's degree; Yuan Li held a bachelor's degree. Jing Tian, Mei Teng, and Yuan Hu held master's degrees. All were registered nurses.
3	Occupation	What was their occupation at the time of the study?	Materials and methods: Data collection / Rigour and reflexivity	The research team were professional nurses. Hong Zheng and Yuan Li were senior clinical nurses; Hong Zheng was head nurse of paediatric cardiology.
4	Gender	Was the researcher male or female?	Materials and methods: Data collection / Data analysis	Hong Zheng, Yuan Li, Jing Tian, and Mei Teng were female; Yuan Hu was male.

5	Experience and training	What experience or training did the researcher have?	Materials and methods: Data collection / Rigour and reflexivity	Both interviewers had formal qualitative-interview training and previous interviewing experience; Hong Zheng had >30 years of paediatric nursing experience.
6	Relationship established	Was a relationship established prior to study commencement?	Materials and methods: Data collection	Neither interviewer had previously participated in the clinical care of the children or families interviewed.
7	Participant knowledge of the interviewer	What did participants know about the researcher?	Materials and methods: Data collection	Participants were informed that interviewers were professional nurses and research-team members and were told the study purpose, voluntary nature, confidentiality arrangements, and intended use of anonymized data.
8	Interviewer characteristics	What characteristics were reported about the interviewer/facilitator?	Materials and methods: Rigour and reflexivity	Reflexive notes considered clinical roles and prompts concerning information overload; the dual interviewer/clinical-reviewer role was explicitly considered.
9	Methodological orientation and theory	What methodological orientation was stated?	Materials and methods: Design and reporting / Analytic scope and data analysis	Qualitative descriptive design and Framework Method.
10	Sampling	How were participants selected?	Materials and methods: Setting, participants, and sample adequacy	Purposive sampling sought variation in education, residence, and treatment pathway.

11	Method of approach	How were participants approached?	Materials and methods: Data collection	Potential participants were identified and contacted through the inpatient ward by Yuan Hu.
12	Sample size	How many participants were in the study?	Results: Participant characteristics	Twelve parents were included in the final analytic sample.
13	Non-participation	How many people refused, withdrew, or were excluded, and why?	Results: Participant characteristics	Sixteen were recruited: one withdrew, two were excluded because diagnoses were outside structural CHD, and one completed interview was excluded before coding because it contained insufficient relevant content.
14	Setting of data collection	Where were data collected?	Materials and methods: Data collection	Face-to-face interviews were conducted individually in a private, quiet room within the department.
15	Presence of non-participants	Was anyone else present?	Materials and methods: Data collection	Interviews were conducted individually; no non-participants were present.
16	Description of sample	What important participant characteristics were reported?	Results: Participant characteristics / Table 1	Parent age, relationship to child, education, residence, child sex, CHD complexity, interview timing, and treatment pathway.

17	Interview guide	Were questions, prompts, and guides provided or pilot tested?	Materials and methods: Data collection / Supplementary File S1	The interview domains and probing approach are described, and the complete semi-structured interview guide is provided. Pilot testing was not reported.
18	Repeat interviews	Were repeat interviews carried out?	Materials and methods: Data collection	No. Each participant completed one interview.
19	Audio/visual recording	Did the research use audio or visual recording?	Materials and methods: Data collection	All interviews were audio-recorded with permission.
20	Field notes	Were field notes made during and/or after interviews?	Materials and methods: Data collection	No formal field notes were taken.
21	Duration	What was the duration of interviews?	Materials and methods: Data collection	Approximately 30 minutes.
22	Data saturation	Was data saturation discussed?	Materials and methods: Setting, participants, and sample adequacy	Sample adequacy was guided by information power. Recruitment ceased when later interviews no longer altered the main analytic categories, theme boundaries, or explanatory relationships.
23	Transcripts returned	Were transcripts returned to participants for comment/correction?	Materials and methods: Rigour and reflexivity	No. Participants could not be recontacted for member checking.
24	Number of data coders	How many data coders coded the data?	Materials and methods: Analytic scope and data analysis	Two researchers (Jing Tian and Mei Teng) coded the Chinese transcripts; unresolved issues were discussed with Yuan Hu.
25	Description of coding tree	Did authors provide a description of the coding tree?	Table 2	Yes. Four themes, 15 subthemes, and their analytic meanings are presented.

26	Derivation of themes	Were themes identified in advance or derived from data?	Materials and methods: Analytic scope and data analysis	Categories, subthemes, and themes were developed from transcript coding within the Framework Method.
27	Software	What software, if applicable, was used to manage the data?	Materials and methods: Analytic scope and data analysis	Coding and construction of the participant-by-domain matrix were completed manually; no qualitative data-analysis software was used.
28	Participant checking	Did participants provide feedback on the findings?	Materials and methods: Rigour and reflexivity	No. Participants could not be recontacted for member checking.
29	Quotations presented	Were participant quotations used and identified?	Results	Yes. Quotations illustrate the themes and are identified using participant codes P1-P12.
30	Data and findings consistent	Was there consistency between data presented and findings?	Results / Table 2	Yes. Themes are supported by explanatory text and participant quotations.
31	Clarity of major themes	Were major themes clearly presented?	Results / Table 2 / Figure 1	Yes. Four major themes are clearly presented.
32	Clarity of minor themes	Were diverse or contradictory cases discussed?	Materials and methods: Rigour and reflexivity / Results / Discussion	Yes. Divergent accounts, including limited searching and information avoidance, were retained and interpreted.
